# A comparison of sexual risk behaviours and HIV seroprevalence among circumcised and uncircumcised men before and after implementation of the safe male circumcision programme in Uganda

**DOI:** 10.1186/s12889-015-2668-3

**Published:** 2016-01-05

**Authors:** Simon Peter Sebina Kibira, Ingvild Fossgard Sandøy, Marguerite Daniel, Lynn Muhimbuura Atuyambe, Fredrick Edward Makumbi

**Affiliations:** 1 Centre for International Health, Department of Global Public Health and Primary Care, Faculty of Medicine and Dentistry, University of Bergen, Bergen, Norway; 2 Department of Community Health and Behavioural Sciences, School of Public Health, Makerere University, Kampala, Uganda; 3 Department of Health Promotion and Development, Faculty of Psychology, University of Bergen, Bergen, Norway; 4 Department of Epidemiology and Biostatistics, School of Public Health, Makerere University, Kampala, Uganda

**Keywords:** Circumcision, Condom use, Survey, Sexual risk behaviours, HIV, Multiple partners, Non-marital sex, Uganda

## Abstract

**Background:**

Although male circumcision reduces the heterosexual HIV transmission risk, its effect may be attenuated if circumcised men increase sexual risk behaviours (SRB) due to perceived low risk. In Uganda information about the protective effects of circumcision has been publicly disseminated since 2007. If increased awareness of the protection increases SRB among circumcised men, it is likely that differences in prevalence of SRB among circumcised versus uncircumcised men will change over time. This study aimed at comparing SRBs and HIV sero-status of circumcised and uncircumcised men before and after the launch of the safe male circumcision programme.

**Methods:**

Data from the 2004 and 2011 Uganda AIDS Indicator Surveys (UAIS) were used. The analyses were based on generalized linear models, obtaining prevalence ratios (PR) as measures of association between circumcision status and multiple sexual partners, transactional sex, sex with non-marital partners, condom use at last non-marital sex, and HIV infection. In addition we conducted multivariate analyses adjusted for sociodemographic characteristics, and the multivariate models for HIV status were also adjusted for SRB.

**Results:**

Twenty six percent of men were circumcised in 2004 and 28 % in 2011. Prevalence of SRB was higher among circumcised men in both surveys. In the unadjusted analysis, circumcision was associated with having multiple sexual partners and non-marital partners. Condom use was not associated with circumcision in 2004, but in 2011 circumcised men were less likely to report condom use with the last non-marital partner. The associations between the other sexual risk behaviours and circumcision status were stable across the two surveys.” In both surveys, circumcised men were less likely to be HIV positive (Adj PR 0.55; CI: 0.41–0.73 in 2004 and Adj PR 0.64; CI: 0.49–0.83 in 2011).

**Conclusions:**

There was higher prevalence of SRBs among circumcised men in both surveys, but the only significant change from 2004 to 2011 was a lower prevalence of condom use among the circumcised. Nevertheless, HIV prevalence was lower among circumcised men. Targeted messages for circumcised men and their sexual partners to continue using condoms even after circumcision should be enhanced to avoid risk compensation.

## Background

Heterosexual transmission of HIV is still the biggest contributor to the HIV epidemic in sub Saharan Africa where over 70 % of the estimated global 35 million HIV positive people live [1, 2]. Male circumcision reduces HIV heterosexual transmission risk from infected women to men [3–8], prevalence of high risk human papilloma virus and incidence of Herpes simplex virus two in men and, genital ulcers in female partners of circumcised HIV negative men [9–12]. In 2007, male circumcision was recommended in 14 sub Saharan African countries with high HIV prevalence but low levels of male circumcision [13, 14].

The Ministry of Health and partners in Uganda have scaled up circumcision through the national safe male circumcision (SMC) programme since 2007. Health workers were provided with accurate information using flip charts and question-answer booklets to assist clients, while media training sessions equipped journalists with information about SMC and its link to HIV prevention. The general public was educated through radio and television talk shows, newspaper columns and educational materials such as brochures for men [15]. A national policy guiding the programme was launched in 2010 [16] together with a national communication strategy [17]. In 2011, there were further social marketing efforts to increase demand, such as the “stand proud, get circumcised” campaign using a provocative approach that spoke to men through women. This was designed to convince men who had intentions of circumcision to get SMC services while encouraging women to support their partners to get circumcised and encouraging adherence to post circumcision practices that promote healing. The SMC intervention is implemented as an additional approach to the existing HIV prevention programmes such promoting condom use and being faithful to one sexual partner, and its demand and service provision increased. Between 2009 and September 2013, over 1.4 million adult men were circumcised [18, 19].

Male circumcision has the potential to reduce the HIV epidemic at population level with large scale benefits projected [20, 21]. There are concerns however that promoting such large scale population level interventions may also come with potential for behavioural risk compensation [22–25]. Circumcised men may as a result of reduced self-perceived risk to HIV and sexually transmitted infections increase sexual risk behaviours, including frequency of unprotected sex with multiple high risk partners [26–28], in part due to misperceptions from social marketing about the ‘partial’ protective effect of male circumcision [29]. Increases in sexual risk behaviours have been documented in Uganda among people living with HIV on antiretroviral therapy [30], partly due to reduced risk perception [31]. HIV vaccine trials have documented similar concerns with increases in sexual risk behaviours after vaccination among some groups [32–34].

Information from the three randomised controlled trials on which the WHO recommendation of the male circumcision intervention was mainly based, indicated both adjustments and non-adjustments in the sexual behaviour of participants. In South Africa [8] circumcised men reported more sexual partners in the 4–21 month recall periods post circumcision while in Kenya [7], inconsistent condom use declined in the control but not in intervention group after a 24 month period of repeated emphasis on comprehensive behaviour related counselling. In contrast, in the Ugandan trial [6, 35], there was no evidence of behavioural risk compensation reported even in follow up studies. However, the authors indicate in the study limitations that all the participants in these studies had received intensive health education and counselling during the trial period, and therefore such results may not be generalizable to the general male population who receive male circumcision through routine services.

There are few studies [23, 28, 36–38] outside of the three trials that have examined the association between male circumcision and sexual risk behaviour. Our earlier analysis of differences in sexual risk behaviours in the 2011 Uganda AIDS indicator survey (UAIS) alone, showed higher odds of engaging in sexual risk behaviours among circumcised men than the uncircumcised [39]. However, no comparison with the period before the implementation of the national SMC programme (2004 UAIS) has been done. The objectives of this paper were to estimate whether there are differences in the associations between sexual risk behaviours and circumcision status, and HIV sero-status and circumcision status between the 2004 and 2011; the periods before and after implementing the SMC programme. We hypothesised a higher prevalence of sexual risk behaviours among circumcised men after information was made public that male circumcision offers partial protection from HIV.

## Methods

### Study design and sampling procedures

This study was based on data from two national surveys; the Uganda HIV/AIDS Sero-Behavioural Survey 2004 (which we refer to as UAIS in this paper) and the UAIS 2011. The 2004 UAIS was conducted before the implementation of the SMC programme while the 2011 UAIS was conducted after the SMC programme implementation was underway in the country. The two surveys have nationally representative samples obtained from stratified two-stage cluster sampling designs [40, 41]. In both surveys, clusters were selected from strata defined by urban/rural residence and geographical regions at the first stage, while the second stage involved selecting households for interview to obtain eligible respondents. Clusters were from a list of enumeration areas obtained from the 2002 Uganda population census (for the 2004 UAIS) and from the 2010 Uganda National Household Survey update of the 2002 Uganda population census (for the 2011 UAIS). At the first stage, 417 clusters in 2004 and 470 in 2011 were selected. The second stage in both surveys involved systematically sampling 25 households for interview in each cluster. Out of 9,842 eligible households, 9,529 were interviewed in 2004 (response rate, 96.1 %) and in these households 8,830 men completed individual interviews out of 9,905 eligible men (response rate, 89.1 %). In the 2011 survey, out of 11,434 occupied households, 11,340 were interviewed, giving a response rate of 99.2 %. In these households 9,588 men were interviewed out of the 9,983 eligible (response rate, 96 %). In both surveys, eligible respondents were permanent residents of the households or visitors who had spent the survey night in the household. All men 15–59 years were requested to voluntarily provide a blood sample for HIV testing. The response rate for HIV testing was 83.4 % in 2004, and 94.2 % in 2011. This paper is based on information from 14,875 men (6,906 in 2004 and 7,969 in 2011 UAIS) who reported to ever have had sex and had information on HIV status.

### Data collection and variables

Data were collected between August 2004 and January 2005 for the 2004 UAIS and between February and September 2011 for the 2011 UAIS. Both surveys were led by the Uganda Ministry of Health working with ICF international, USA and Uganda Bureau of Statistics. Individual male interviews obtained data on respondents’ self-reported circumcision status, their reported sexual behaviours, personal perceived risk of HIV infection, and knowledge of the protection offered by male circumcision against HIV infection (for 2011 alone), and socio-demographic characteristics (age, marital status, highest education level, survey region, ethnicity, residence, religion). Information on wealth status was also obtained from the household interviews and thus reflects the state of the household in which individual men were interviewed. All male interviews were conducted by trained male research assistants.

Laboratory technicians collected blood samples (venous blood or dried blood spots for those who refused venous blood draw) for HIV testing. Tests for the both surveys were conducted at a central laboratory of the Uganda Virus Research Institute using Murex HIV 1.2.0 (Abbott) assay. Samples that were HIV-reactive with Murex were re-tested with Vironostika HIV Uni-Form II Plus-O to confirm their sero-status, while ANILAB Systems HIV enzyme immunoassay was used to resolve discordant results. All the positive specimens and 5 % of the negative specimens were re-tested at the Centers for Disease Control laboratory in Uganda using the same testing algorithm, for quality control purposes. Further details on the tests and quality control are available in the main survey reports [40, 41].

The dependent variables were HIV sero-status obtained from blood sample tests in both surveys, and the following sexual risk behaviours [42] among sexually active circumcised and uncircumcised men: (a) having multiple sexual partners, (b) having had sex with non-marital partners, (c) non-use of condoms at the last non-marital sex, and (d) transactional sex (payment or receipt of money/gifts in exchange for sex). All these questions referred to behaviours that took place in the 12 months preceding each of the surveys. Condom use at last non-marital sex only included men who reported having such sex. The main independent variable was self-reported circumcision status, while other explanatory variables were socio-demographic characteristics, personal HIV risk perception as well as knowledge of the protection offered by male circumcision against HIV infection (for the 2011 UAIS).

### Statistical analyses

Analyses were conducted using Stata version 13 (StataCorp 2013). Men’s individual data files were sorted by unique identifiers to link them to the HIV sero-status data for each survey. Data from the two national surveys were then appended to get one dataset with 14,875 observations. A “survey” variable was generated to identify each of the surveys’ datasets.

The measure of association used for these analyses were prevalence ratios (PR) and their corresponding 95 % confidence intervals [43–45] obtained via modified Poisson regression models using generalized linear models with family (Poisson) and link (log). First we estimated the associations between male circumcision and sexual risk behaviours, or male circumcision and HIV sero-status for each of the surveys. In the adjusted analyses of sexual risk behaviour, socio-demographic characteristics were controlled for. When there was 10 % difference in the survey specific PRs, an interaction term between male circumcision and year of the survey was introduced in combined regression models for each of the sexual risk behaviours to further test if the survey period was important. Sample weights were used in the analyses. We also adjusted for clustering of observations within the same cluster by use of the cluster command in Stata.

### Ethical considerations

Informed consent was obtained before conducting interviews, and separate consent was obtained for taking blood samples. For confidentiality purposes, all personal information that could potentially identify an individual (such as name and address) was destroyed before linking that HIV data to the socio-demographic and behavioural data collected in the individual questionnaires. In the 2004 survey, HIV test results were not provided from the survey but the respondents who wanted to know their HIV status were given a voucher for a free voluntary counselling and testing visit at a nearby health facility or an outreach point established by the survey project [41]. In 2011, home based rapid HIV testing was done and test results were provided on the same day for respondents who wanted to receive them, in addition to the central laboratory tests. Those who tested positive were told to obtain CD4 results six weeks after the interview at a nearby health facility. Counselling was also provided before and after testing by trained counsellors for those who opted to receive results [40].

Each survey protocol was reviewed and approved by the Science and Ethics Committee of the Uganda Virus Research Institute, ICF International’s Institutional Review Board, and a review committee at the Centers for Disease Control and Prevention in Atlanta, USA. They were also cleared by the Ethics Committee of the Uganda National Council for Science and Technology. Permission to use both surveys’ data was obtained from ICF international, USA, and the Ministry of Health, Uganda.

## Results

### Characteristics of respondents

A total of 14,875 men were analysed in the two surveys. We excluded 531 men from the analysis for this study; sixteen men in 2004 and one in the 2011 survey had indeterminate HIV test results, a further 458 men in 2004 and 51 in 2011 had missing HIV results, and five men in 2004 had missing circumcision status data.

In total 1,792 (26 %) and 2,228 (28 %) men reported that they were circumcised in 2004 and 2011, respectively. In 2004, two thirds (67 %) of men were married and 86 % lived in rural areas, while in 2011, 72 % were married and 81 % lived in rural areas. The majority (61 % in 2004 and 57 % in 2011) of the men had completed primary education but a higher proportion in 2011 (36 %) had completed secondary or higher education than in 2004 (29 %). In both surveys, 44 % were from households in the top two wealth quintiles, and the largest ethnic groups were Baganda, Banyankore and Langi/Acholi. Two thirds (65 %) in 2011 perceived themselves as being at high risk for HIV and 50 % knew that male circumcision reduced the risk of HIV infection to a man.

In 2004, over half of circumcised men (53 %) were from households in the top two wealth quintiles compared to only 44 % of the uncircumcised. Circumcised men were also more educated and more likely to be from urban areas than their uncircumcised counterparts in both surveys. Among the uncircumcised men, 6.8 % in 2004 and 7.8 % in 2011 tested positive for HIV while among the circumcised, 4.3 and 4.8 % in 2004 and 2011, respectively, tested positive.

In 2011, a larger proportion of circumcised than uncircumcised men knew that circumcision was protective (62 % against 46 %), but the personal perception of HIV risk was similar across both groups (64 % among circumcised, 66 % among the uncircumcised) (Table 1).

**Table 1:** Characteristics of circumcised and uncircumcised men 15–59 years, Uganda 2004 and 2011

Variables	2004 UAIS, n (%)			2011 UAIS, n (%)		
	Circumcised	Uncircumcised	All men	Circumcised	Uncircumcised	All men
Age						
15–24	492 (27.4)	1,318 (25.8)	1,809 (26.2)	610 (27.4)	1,331 (23.2)	1,941 (24.4)
25–34	549 (30.6)	1,664 (32.6)	2,213 (32.1)	708 (31.8)	1,751 (30.5)	2,460 (30.9)
35–44	434 (24.2)	1,162 (22.7)	1,596 (23.1)	508 (22.8)	1,492 (26.0)	2,000 (25.1)
45–59	317 (17.7)	970 (19.0)	1,288 (18.6)	402 (18.0)	1,166 (20.3)	1,568 (19.7)
Marital status						
Never married	418 (23.3)	1,170 (22.9)	1,589 (23.0)	523 (23.5)	1,127 (19.6)	1,649 (20.7)
Married	1,183 (66.0)	3,438 (67.2)	4,621 (66.9)	1,534 (68.9)	4,176 (72.7)	5,710 (71.7)
Divorced/Widowed	191 (10.6)	506 (9.9)	696 (10.1)	171 (7.7)	438 (7.6)	609 (7.7)
Residence						
Urban	352 (19.6)	605 (11.8)	957 (13.9)	604 (27.1)	916 (16.0)	1,520 (19.1)
Rural	1,440 (80.4)	4,509 (88.2)	5,949 (86.2)	1,624 (72.9)	4,825 (84.1)	6,449 (80.9)
Region						
Central	468 (26.1)	1,213 (23.7)	1,681 (24.4)	491 (22.0)	1,293 (22.5)	1,784 (22.4)
Kampala	332 (18.6)	645 (12.6)	978 (14.2)	215 (9.7)	353 (6.2)	568 (7.1)
Eastern	465 (25.9)	817 (16.0)	1,282 (18.6)	882 (39.6)	819 (14.3)	1,701 (21.4)
Northern	458 (25.6)	1,712 (33.5)	2,171 (31.4)	201 (9.0)	1,798 (31.3)	1,999 (25.1)
Western	69 (3.8)	725 (14.2)	794 (11.5)	439 (19.7)	1,477 (25.7)	1,916 (24.1)
Highest Education Level						
No Education	164 (9.1)	529 (10.4)	693 (10.1)	143 (6.4)	427 (7.4)	570 (7.2)
Primary	1,058 (59.1)	3,167 (62.0)	4,225 (61.3)	1,166 (52.3)	3,360 (58.5)	4,526 (56.8)
Secondary	442 (24.7)	1,066 (20.9)	1,509 (21.9)	697 (31.3)	1,458 (25.4)	2,155 (27.0)
Tertiary	125 (7.0)	342 (6.7)	468 (6.8)	222 (10.0)	496 (8.6)	718 (9.0)
Wealth level						
Low	496 (27.7)	1,999 (39.1)	2,495 (36.1)	654 (29.4)	2,297 (40.0)	2,952 (37.0)
Middle	347 (19.4)	1,012 (19.8)	1,359 (19.7)	428 (19.2)	1,103 (19.2)	1,531 (19.2)
High	949 (52.9)	2,103 (41.1)	3,052 (44.2)	1,146 (51.4)	2,341 (40.8)	3,486 (43.8)
Ethnicity						
Baganda	357 (19.9)	785 (15.4)	1,142 (16.6)	400 (18.0)	921 (16.1)	1,321 (16.6)
Banyakore	68 (3.8)	606 (11.9)	674 (9.8)	109 (4.9)	685 (11.9)	793 (10.0)
Iteso/Karimojong	47 (2.6)	621 (12.2)	668 (9.7)	64 (2.9)	667 (11.6)	730 (9.2)
Lugbara/Madi	184 (10.3)	292 (5.7)	477 (6.9)	113 (5.1)	282 (4.9)	396 (5.0)
Basoga	217 (12.1)	416 (8.1)	632 (9.2)	314 (14.1)	401 (7.0)	716 (9.0)
Langi/Acholi	21 (1.2)	765 (15.0)	786 (11.4)	19 (0.9)	877 (15.3)	896 (11.2)
Bakiga/Bafumbira	45 (2.5)	434 (8.5)	479 (7.0)	66 (2.9)	526 (9.2)	592 (7.4)
Bagisu/Sabiny/Bakonzo	395 (22.0)	54 (1.1)	449 (6.5)	646 (29.0)	34 (0.6)	680 (8.5)
Alur/Japadhola	76 (4.2)	321 (6.3)	397 (5.8)	73 (3.3)	315 (5.5)	387 (4.9)
Banyoro/Batooro	81 (4.5)	323 (6.3)	404 (5.9)	164 (7.4)	516 (9.0)	680 (8.5)
Others	300 (16.8)	488 (9.6)	788 (11.4)	261 (11.7)	516 (9.0)	777 (9.8)
Religion						
Non Moslem	931 (52.0)	5,085 (99.8)	6,016 (87.4)	1,202 (54.0)	5,729 (99.8)	6,931 (87.0)
Moslem	858 (48.0)	12 (0.2)	870 (12.6)	1,026 (46.1)	12 (0.2)	1,038 (13.0)
Perceived HIV risk						
Low risk				743 (33.4)	1,721 (30.0)	2465 (30.9)
High risk/not sure				1,431 (64.2)	3,772 (65.7)	5202 (65.3)
Missing				54 (2.4)	248 (4.3)	302 (3.8)
Knows circumcision Reduces HIV risk						
No				826 (37.1)	3,029 (52.8)	3855 (48.4)
Yes				1,389 (62.4)	2,634 (45.9)	4023 (50.5)
Missing				13 (0.6)	78 (1.4)	91 (1.1)
HIV sero-status						
Negative	1,716 (95.7)	4,767 (93.2)	6,482 (93.9)	2,120 (95.2)	5,296 (92.3)	7,416 (93.1)
Positive	76 (4.3)	347 (6.8)	424 (6.1)	108 (4.8)	445 (7.8)	553 (6.9)
Total	1,792 (100)	5,114 (100)	6,906 (100)	2,228 (100)	5,741 (100)	7,969 (100)

### Prevalence of sexual risk behaviours

The prevalence of multiple and non-marital sexual partnerships was stable over the two survey periods. In the 2004 survey, 25 % of men reported sex with multiple partners while in 2011, 22 % reported this behaviour. Thirty five percent of men reported sex with a non-marital partner in 2004 compared to 33 % in 2011. However, the percentage of men who reported non-use of condoms at the last such sexual intercourse was higher in the 2011 survey (55 % compared to 48 % in 2004). There was an increase in the proportion of men who reported transactional sex from 1.2 % in 2004 to 2.7 % in 2011 (Table 2).

**Table 2:** Prevalence of Sexual risk behaviours among circumcised and uncircumcised men, Uganda 2004 and 2011

Variables	2004 UAIS, n (%)			2011 UAIS, n (%)		
	Circumcised	Uncircumcised	All men	Circumcised	Uncircumcised	All men
Had multiple sexual partners						
No	1,201 (67.0)	3,996 (78.1)	5,196 (75.2)	1,615 (72.5)	4,572 (79.7)	6,187 (77.6)
Yes	592 (33.0)	1,118 (21.9)	1,710 (24.8)	613 (27.5)	1,168 (20.4)	1,781 (22.4)
Total	1,792 (100)	5,114 (100)	6,906 (100)	2,228 (100)	5,741 (100)	7,969 (100)
Had transactional sex						
No	1,761 (98.2)	5,063 (99.0)	6,824 (98.8)	2,154 (96.7)	5,601 (97.6)	7,755 (97.3)
Yes	31 (1.8)	51 (1.0)	82 (1.2)	74 (3.3)	139 (2.4)	214 (2.7)
Total	1,792 (100)	5,114 (100)	6,906 (100)	2,228 (100)	5,741 (100)	7,969 (100)
Sex with a non-marital partner						
No	926 (59.3)	2,951 (67.8)	3,878 (65.5)	1,229 (61.6)	3,569 (69.8)	4,798 (67.5)
Yes	636 (40.7)	1,404 (32.2)	2,040 (34.5)	768 (38.5)	1,547 (30.2)	2,315 (32.6)
Total	1,562 (100)	4,355 (100)	5,918 (100)	1,997 (100)	5,116 (100)	7,114 (100)
Used a condom at last non marital sex						
No	290 (45.6)	692 (49.3)	983 (48.2)	448 (58.4)	819 (52.9)	1,267 (54.7)
Yes	346 (54.4)	711 (50.7)	1,057 (51.8)	320 (41.6)	728 (47.1)	1,048 (45.3)
Total	636 (100)	1,404 (100)	2,040 (100)	768 (100)	1,547 (100)	2,315 (100)

### Sexual risk behaviour differences between circumcised and uncircumcised men in 2004 and 2011

The prevalence of all sexual risk behaviour was higher among the circumcised than the uncircumcised men in both survey periods (Table 2). When we adjusted for socio-demographic variables, circumcision status was significantly associated with having multiple sexual partners both in 2004 and 2011 (2004: adjusted PR 1.38; 95 % CI 1.26–1.51]; 2011: adjusted PR 1.23; 95 % CI 1.11–1.36), and having had sex with non-marital sexual partners in 2004 (adjusted PR 1.12; 95 % CI 1.06–1.20) in the 12 months preceding each survey. There was no difference in 2004 between the two groups regarding condom use at last non-marital sex. However, in 2011, circumcised men were less likely to report use of condoms at the last sex with a non-marital partner than uncircumcised men (adjusted PR 0.85; CI 0.76–0.96). Male circumcision status was not significantly associated with transactional sex in any of the two surveys. Other factors independently associated with sexual risk behaviours were age, marital status, education level, region of residence and wealth quintile of the man’s household (Table 3).

**Table 3:** Generalised linear models showing unadjusted and adjusted associations between sexual risk behaviours and circumcision status among men age 15–59 years, Uganda 2004 and 2011

	Had multiple sexual partners in last 12 months, PR [95 % CI]		Had sex with non-marital partner in last 12 months, PR [95 % CI]		Used a condom at last non marital sex, PR [95 % CI]		Transactional sex in last 12 months, PR [95 % CI]	
	2004	2011	2004	2011	2004	2011	2004	2011
Unadjusted: Circumcised								
No	1.0	1.0	1.0	1.0	1.0	1.0	1.0	1.0
Yes	1.51* [1.38,1.65]	1.35* [1.23,1.49]	1.26* [1.17,1.37]	1.27* [1.17,1.38]	1.07 [0.98,1.18]	0.88* [0.79,0.99]	1.72 [1.06,2.81]	1.36 [0.99,1.88]
Adjusted^a^: Circumcised								
No	1.0	1.0	1.0	1.0	1.0	1.0	1.0	1.0
Yes	1.38* [1.26,1.51]	1.23* [1.11,1.36]	1.12* [1.06,1.20]	1.05 [0.99,1.13]	1.00 [0.92,1.10]	0.85* [0.76,0.96]	1.56 [0.92,2.62]	1.23 [0.85,1.76]
Number of men	6886	7857	5919	6996	1945	2233	6886	7857

^a^Adjusted for highest education level, Age, Marital status, Survey region, Residence, and Wealth status

*p < 0.05

There was interaction between the effect of circumcision and age on transactional sex. There was also interaction between the effect circumcision and age on non-marital sex. Circumcision was more strongly associated with these transactional sex among the older (25–59 years) than the younger men (15–24 years). This was also similar for men reporting non-marital sex. Interaction between the effect of circumcision and urban/rural residence on transactional sex was also observed. A slightly higher proportion of circumcised men in the rural areas reported engaging in transactional sex in 2011 than in the 2004 survey. A similar trend was observed among uncircumcised men in urban areas. These stratified results are however based on very few men reporting transactional sex.

The models with combined data from the two surveys with an interaction term for “circumcision status and survey period” indicate that non-use of condoms at the last non-marital sex among circumcised men significantly varied by survey. There was a reduction in condom use in 2011, with circumcised men significantly less likely to report use. The association between circumcision and multiple sexual partners did not significantly vary between 2004 and 2011 (Table 4).

**Table 4:** Models of the associations between sexual risk behaviours and circumcision status with combined data from the 2004 and 2011 UAIS

	Had multiple sexual partners in last 12 months		Used a condom at last non marital sex	
	Unadjusted,	Adjusted,	Unadjusted,	Adjusted,
	PR [95 % CI]	PR [95 % CI]	PR [95 % CI]	PR [95 % CI]
Circumcised^a^				
No	1.0	1.0	1.0	1.0
Yes	1.51* [1.37,1.66]	1.42* [1.29,1.56]	1.07 [0.96,1.20]	1.02 [0.93,1.12]
Survey				
2004 UAIS	1.0	1.0	1.0	1.0
2011 UAIS	0.93 [0.84,1.03]	0.92 [0.83,1.02]	0.93 [0.84,1.03]	0.99 [0.91,1.07]
Interaction term (circumcision and survey)	0.90 [0.77,1.04]	0.89 [0.77,1.03]	0.82* [0.70,0.97]	0.81* [0.71,0.93]
Number of men	14757	14743	4181	4178

^a^Adjusted for highest education level, Age, Marital status, Survey region, Residence, and Wealth status

*p < 0.05

### Male circumcision and HIV sero-status

Male circumcision was significantly associated with lower HIV prevalence across both surveys. After adjusting for background characteristics, circumcised men were 43 % less likely to test HIV positive in 2004 (adjusted PR 0.57; CI 0.44–0.75) and 34 % less likely in the 2011 UAIS (adjusted PR 0.66; CI 0.51–0.84) compared to the uncircumcised. The PRs did not change substantially after including sexual risk behaviours in the models (Table 5).

**Table 5:** Generalised linear models showing unadjusted and adjusted associations between circumcision status and HIV test results among circumcised and uncircumcised men age 15–59 years, Uganda 2004 and 2011

	(1)	(2)	(3)	(4)	(5)	(6)
	Unadjusted: Tested HIV positive, 2004 PR (95 % CI)	Unadjusted: Tested HIV positive, 2011 PR (95 % CI)	Adjusted for background characteristics^a^: Tested HIV positive, 2004 PR (95 % CI)	Adjusted for background characteristics^a^: Tested HIV positive, 2011 PR (95 % CI)	Adjusted for background characteristics and sexual risk behaviours^b^: Tested HIV positive, 2004 PR (95 % CI)	Adjusted for background characteristics and sexual risk behaviours^b^: Tested HIV positive, 2011 PR (95 % CI)
Circumcised						
No	1.0	1.0	1.0	1.0	1.0	1.0
Yes	0.63* [0.48,0.82]	0.62* [0.49,0.80]	0.57* [0.44,0.75]	0.66* [0.51,0.84]	0.55* [0.41,0.73]	0.64* [0.49,0.83]
Number of men	6900	7857	6886	7857	5919	6996

^a^Adjusted for highest education level, Age, Marital status, Survey region, Residence, and Wealth status

^b^Adjusted for highest education level, Age, Marital status, Survey region, Residence, and Wealth status, Multiple sexual partners, Sex with a non-marital partner, Transactional sex

*p < 0.05

## Discussion

Circumcised men reported higher prevalence of all sexual risk behaviours examined, except for transactional sex, than uncircumcised men. Use of condoms with the last non-marital sexual partner among circumcised men reporting non-marital sex was lower in 2011 compared with 2004. However, there was no significant change in the prevalence of other sexual risk behaviours between the two survey periods. Thus we conclude that there is limited evidence to support our hypothesis from the two UAISs. Even with higher reported prevalence of sexual risk behaviours, circumcised men were less likely to test HIV positive than the uncircumcised in both surveys.

It is plausible that the reduction in condom use among circumcised men could be linked to risk compensation due to higher awareness in 2011 that circumcision was protective since a similar reduction in reported condom use at the last non-marital sex was not found among uncircumcised men. Since condoms are even more effective against heterosexual HIV infection than circumcision [46, 47], a reduction in their use because of male circumcision [48] would be a dangerous ‘trade off’. Inconsistent condom use after circumcision has been associated with increased risk of HIV infection among young men in eastern Uganda [49]. This could significantly reduce the beneficial effect of circumcision against HIV infection, even with its reported high efficacy levels [21, 50].

Circumcised men reported higher prevalence of multiple sexual partners in both 2004 and 2011 than the uncircumcised. Although there were no significant differences in the association over time, i.e. indicating that any risk compensation due to the SMC campaign was limited at this early stage of the campaign, multiple sexual partnerships coupled with higher prevalence of non-use of condoms in 2011 is a potentially dangerous situation if it continues uncontrolled. If persons who have multiple sexual relationships also have concurrent partners, non-use of condoms is particularly risky because HIV infection can easily spread to several persons in the sexual network if one of the concurrent partners are newly infected (and thus more infectious) [51, 52]. Concurrency has been one of the main drivers of heterosexual HIV infections in sub Saharan Africa in the past decades [1, 53, 54].

Further, because of the early stages of the SMC campaign, it is possible that some previously circumcised men may not have fully understood partial risk reduction as opposed to eliminating the entire risk of HIV infection, leading to a misguided sense of sexual freedom [48]. These two concepts may still be hard for the population to understand fully even in the current stage of the campaign, a challenge that could further be complicated by appropriate translation into all local dialects for diverse populations ([55], p.26). It may be hard to convince all circumcised men as well as their sexual partners to continue using condoms after circumcision, even when engaging in high risk behaviours such as multiple sexual partnerships. However, if such behaviour continues unabated in the current ‘mature’ period of the SMC programme, this should have implications for circumcision-related social marketing messages that mainly focus on those intending to circumcise, and less on behaviours of men already circumcised.

Although a higher occurrence of sexual risk behaviours was reported among circumcised men, the HIV prevalence was significantly lower among this group than the uncircumcised in both 2004 and 2011 survey. The associations remained significant even after adjusting for sexual risk behaviours in the final model. Higher sexual risk behaviours among circumcised men did not seem to affect their HIV risk. This further supports the evidence for protection that male circumcision offers against HIV infection [3, 6–8]. However, caution needs to be consistently publicly re-echoed to ensure circumcised men embrace safer sexual behaviours even with knowledge that the intervention is protective.

The study has several limitations. First, the cross sectional nature of both surveys means inability to ascertain temporality and causation between circumcision, sexual behaviour and HIV status. Second, both circumcision status and the sexual risk behaviours were obtained using individual men’s self-reports in face-to-face interviews which can be liable to social desirability [56] as well as recall biases when reporting for a 12 months periods. However, all the individual interviews were conducted by well-trained male interviewers using standardised questionnaires. The results from this study are from nationally representative samples of men with a high response rate and can be generalised to the general adult male population in Uganda. The surveys are also drawn using the same standard sampling methodology from a similar target population 5 years apart. Even though they are not panel surveys, they can be comparable across the time points.

## Conclusions

This study indicates higher prevalence of sexual risk behaviours among circumcised men in each survey and lower prevalence in use of condoms with non-marital sexual partners among circumcised men in 2011, suggesting possible risk compensation among some circumcised men. However, even with higher prevalence of sexual risk behaviours, circumcised men still had significantly lower HIV prevalence than their uncircumcised counterparts. Considering the high levels of sexual risk behaviours among men who are already circumcised observed in this study, the Ministry of Health and partners need to continue sensitising the sexually active population to use condoms especially when having multiple sexual partners, even when a man is circumcised. These messages should target both circumcised men and their sexual partners. Educating men undergoing circumcision also needs to be strengthened to avoid sexual risk taking post circumcision.
